# Retinoic Acid Receptor-Dependent, Cell-Autonomous, Endogenous Retinoic Acid Signaling and Its Target Genes in Mouse Collecting Duct Cells

**DOI:** 10.1371/journal.pone.0045725

**Published:** 2012-09-26

**Authors:** Yuen Fei Wong, Patricia D. Wilson, Robert J. Unwin, Jill T. Norman, Matthew Arno, Bruce M. Hendry, Qihe Xu

**Affiliations:** 1 Department of Renal Medicine, King’s College London, London, United Kingdom; 2 Centre for Nephrology, University College London, London, United Kingdom; 3 Genomics Centre, King’s College London, London, United Kingdom; INSERM, France

## Abstract

**Background:**

Vitamin A is necessary for kidney development and has also been linked to regulation of solute and water homeostasis and to protection against kidney stone disease, infection, inflammation, and scarring. Most functions of vitamin A are mediated by its main active form, all-*trans* retinoic acid (tRA), which binds retinoic acid receptors (RARs) to modulate gene expression. We and others have recently reported that renal tRA/RAR activity is confined to the ureteric bud (UB) and collecting duct (CD) cell lineage, suggesting that endogenous tRA/RARs primarily act through regulating gene expression in these cells in embryonic and adult kidney, respectively.

**Methodology/Principal Findings:**

To explore target genes of endogenous tRA/RARs, we employed the mIMCD-3 mouse inner medullary CD cell line, which is a model of CD principal cells and exhibits constitutive tRA/RAR activity as CD principal cells do in vivo. Combining antagonism of RARs, inhibition of tRA synthesis, exposure to exogenous tRA, and gene expression profiling techniques, we have identified 125 genes as candidate targets and validated 20 genes that were highly regulated (Dhrs3, Sprr1a, and Ppbp were the top three). Endogenous tRA/RARs were more important in maintaining, rather than suppressing, constitutive gene expression. Although many identified genes were expressed in UBs and/or CDs, their exact functions in this cell lineage are still poorly defined. Nevertheless, gene ontology analysis suggests that these genes are involved in kidney development, renal functioning, and regulation of tRA signaling.

**Conclusions/Significance:**

A rigorous approach to defining target genes for endogenous tRA/RARs has been established. At the pan-genomic level, genes regulated by endogenous tRA/RARs in a CD cell line have been catalogued for the first time. Such a catalogue will guide further studies on molecular mediators of endogenous tRA/RARs during kidney development and in relation to renal defects associated with vitamin A deficiency.

## Introduction

All-*trans* retinoic acid (tRA) is the primary bioactive form of endogenous retinoids derived from dietary vitamin A and plays important roles in regulating a myriad of physiological events [1]. One of the major mechanisms through which tRA exerts its biological activity is by binding and activating its cognate nuclear receptors, the retinoic acid receptors (RARs) α, β, γ, and retinoid X receptors (RXRs) α, β, γ, which heterodimerize to act as transcription factors, thereby modulating gene transcription [2]. Direct target genes of tRA are often characterized by the presence of one or more retinoic acid response elements (RAREs) in the gene regulatory region, which serve as anchorage points for the RXR-RAR heterodimers [3], [4]. A RARE typically consists of two direct repeats (DR) of the hexameric motif PuG(G/T)TCA, separated by 1, 2, or 5 nucleotides, referred to as DR1, DR2, and DR5, respectively [2]. While DR5 RAREs represents the most potent classical RARE for transcriptional activity regulation, other forms of non-classical RAREs, e.g., hexameric motifs spaced by more than 5 nucleotides, and imperfect hexameric motifs have been described [3]. Other than the canonical tRA/RAR/RARE signaling, various non-canonical signaling events of retinoids, such as RAR-independent signaling and the involvement of ligands other than tRA, have been described [5]. In addition to the multiplicity of retinoid signaling, the presence of endogenous retinoids is also intricately controlled by multiple synthesizing and metabolizing enzymes, including medium-chain and short-chain dehydrogenases, retinaldehyde dehydrogenases (Raldhs), and the cytochrome P450 family 26 [6]. It is thus not surprising that retinoid signaling is highly complex, reflected in its diverse and seemingly paradoxical effects, depending on different cell types and different settings.

It is well established that the endogenous tRA and RARs are indispensable for embryonic kidney development [7]. Even mild gestational vitamin A deficiency leads to a deficit in nephron number, which may predispose the kidney to abnormal development or function when associated with other morbidities [8]. Using *RARE-hsp68-lacZ* mice as a reporter model, we recently described the presence of a RARE reporter signal indicative of RAR-dependent tRA activity in the collecting ducts (CDs) of young and adult mouse kidneys [9]. Our observation of this RARE reporter signal in the CDs is similar to the finding of Rosselot et al., who described the presence of a RARE reporter signal in the ureteric buds (UBs) of embryonic kidney, which are the embryonic precursors of CDs [10]. Thus, RARE activity in healthy kidneys appears to be confined to the UB/CD cell lineage.

The observation of RARE reporter activity in the UB/CD cell lineage has important implications. Initially Batourina et al proposed that kidney stromal mesenchyme is the primary site of action for tRA/RAR signaling in embryonic kidneys; the signal being delivered to the UB to regulate Ret expression, which stimulates ureteric bud branching and initiates kidney development [11]. The same group recently revised this model and proposed that tRA/RAR signaling was actually initiated in the UB cells rather than in the stromal mesenchyme [10]. The revised model is in good concordance with the presence of a RARE reporter signal in UB cells. Nevertheless, tRA/RAR target genes in UB cells remain largely unknown, as are the tRA/RAR target genes in UB-derived CDs. Because RARs are nuclear transcription factors known to regulate gene transcription, and in view of the continuity of tRA/RAR signaling in the UB/CD cell lineage in embryonic and postnatal kidneys [9], [10], it is relevant to explore what genes are under the control of the endogenous tRA/RARs in CD cells.

**Figure 1:**
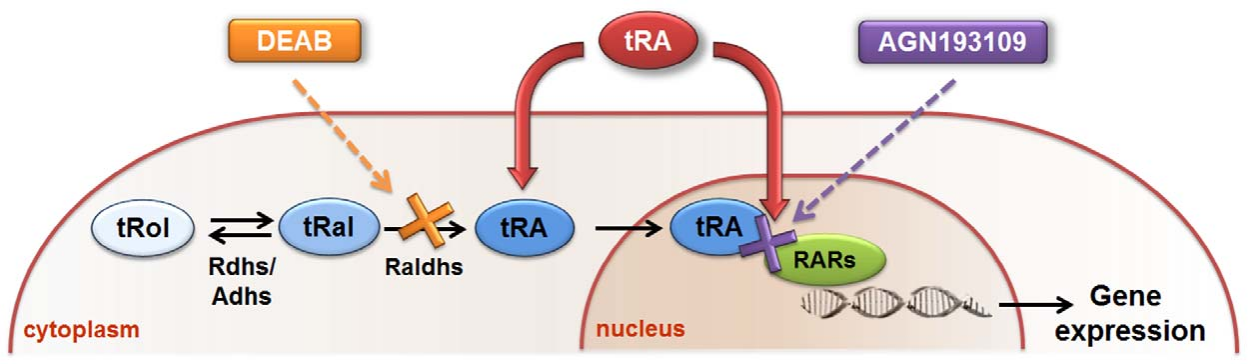
Strategy employed to examine endogenous tRA/RAR signaling and its target genes. In cells, all-*trans* retinol (tRol), i.e., vitamin A, is oxidized reversibly to all-*trans* retinaldehyde (tRal), catalyzed by the enzymes retinol dehydrogenases (Rdhs) or alcohol dehydrogenases (Adhs). tRal is then oxidized irreversibly to all-*trans* retinoic acid (tRA), catalyzed by retinaldehyde dehydrogenases (Raldhs). tRA translocates into cell nucleus to bind and activate retinoic acid receptors (RARs), modulating gene transcription. AGN193109 (purple) and 4-(diethylamino)benzaldehyde (DEAB, orange) were used to compete with tRA for RAR binding and to inhibit Raldhs in the conversion of tRal into tRA, respectively. Specificity of inhibition was confirmed by adding exogenous tRA (red) simultaneously to examine if the effects of AGN193109 and DEAB could be at least partially abolished.

To explore target genes of endogenous tRA/RARs in the UB/CD cell lineage, we performed microarray experiments using mIMCD-3 as an *in vitro* cell model. mIMCD-3 is a well-characterized inner medullary CD cell line [12], which has been widely used in a variety of studies to explore the functions and activities of UB/CD cells: ion channel signaling [13], [14], signaling during osmotic and hypertonic stress [15], urea signaling [16], and branching morphogenesis [17]. We used two different chemicals to disrupt endogenous tRA/RAR signaling at different stages: (i) AGN193109 [18], a pan-antagonist of RARs, which competes with endogenous tRA for RAR binding, and (ii) 4-(diethylamino)benzaldehyde (DEAB) [19], an inhibitor of Raldhs, which inhibits endogenous tRA biosynthesis. To confirm the specificity of these chemical reagents, exogenous tRA was added simultaneously to determine if their effects could be abolished (Figure 1).

## Results

### The mIMCD-3 Cell Line Simulates CD Cells *in vivo*, Expressing Epithelial and CD Principal Cell Markers and Demonstrating Constitutive tRA/RAR Activity

**Figure 2:**
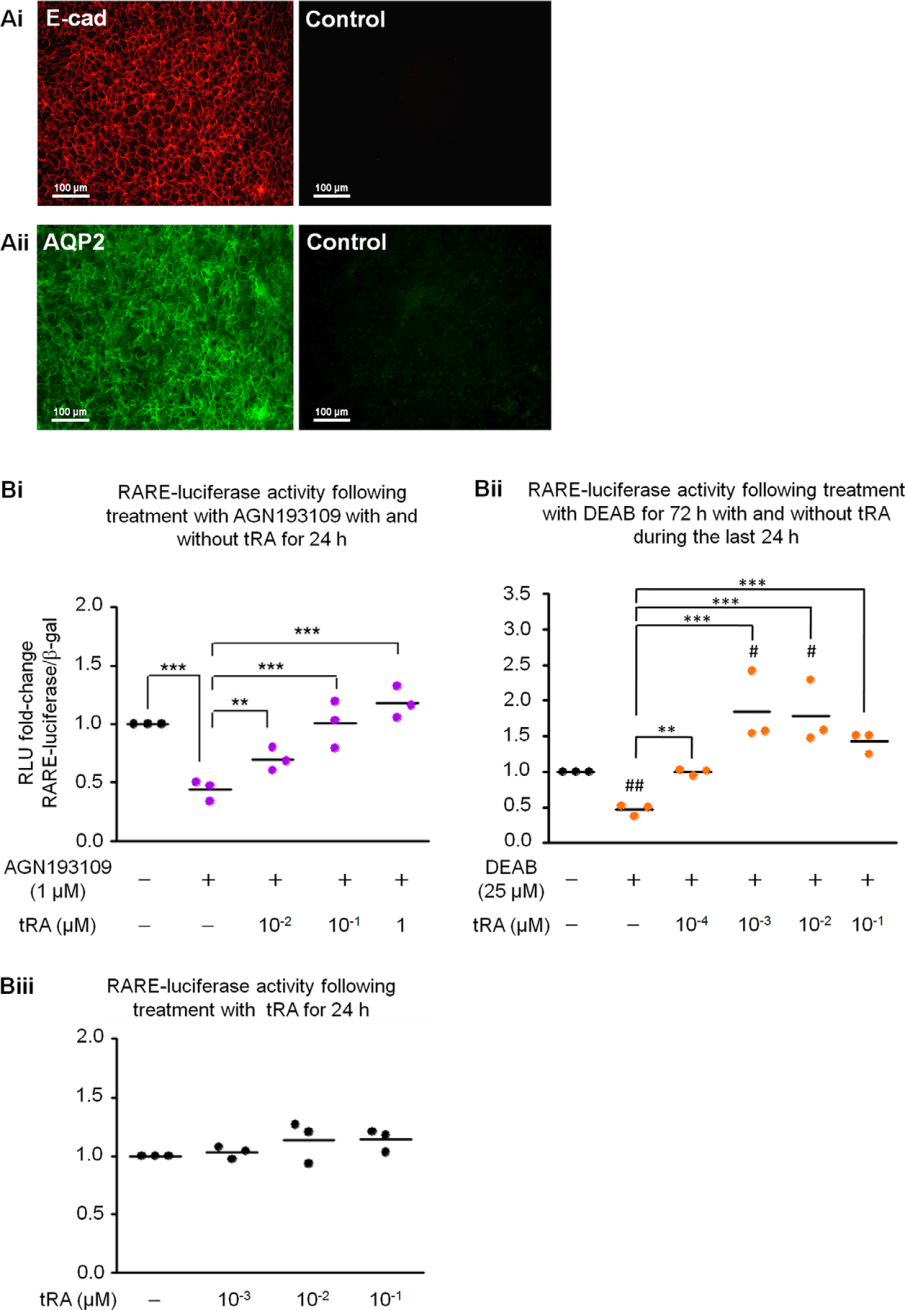
mIMCD-3 cells as an *in vitro* model to examine target genes of endogenous tRA/RARs. mIMCD-3 cells expressed E-cadherin (E-cad) (**Ai**) and aquaporin 2 (AQP2) (**Aii**) proteins. Left panels showed positive staining of specific antibodies, whereas right panels showed negative controls of cells stained with the respective non-immune IgGs. Original magnification was 200×. **Bi.** Treating cells with 1 µM AGN193109 for 24 h resulted in a decrease of RARE-luciferase activity to about 50% of that of the vehicle control group; the reduction was at least partially abolished with simultaneous addition of exogenous tRA at 0.01–1 µM, in a dose-dependent manner. ** and ***: p<0.01 and p<0.001, respectively. **Bii.** Treating cells with 25 µM DEAB for 72 h resulted in a suppression of RARE-luciferase activity to about 50% of that of vehicle control group; when exogenous tRA was added during the last 24 h, the suppression of RARE-luciferase activity was reversed in a dose-dependent manner, saturated at 1 nM tRA. # and ##: p<0.05 and 0<0.01 vs vehicle control group, respectively; ** and ***: p<0.01 and p<0.001 vs DEAB-only group, respectively. **Biii.** When cells were treated with exogenous tRA alone at 0.001–0.1 µM for 24 h, a slight trend of dose-dependent increase of RARE-luciferase activity was noted but the difference was not statistically significant. Each dot represents mean value of triplicates or quadruplicates from a single biological experiment.

mIMCD-3 cells expressed E-cadherin, a marker of epithelial cells (Figure 2Ai), and aquaporin 2, a marker of CD principal cells (Figure 2Aii), confirming its epithelial and CD-like phenotype. After confirming the identity of mIMCD-3 cells, we examined the presence of RARE-reporter activity by transfecting the cells with pGL3-RARE-luciferase reporter plasmid. By treating mIMCD-3 cells with AGN193109, basal RARE-luciferase activity was reduced to about 50% of that of the vehicle control group; when exogenous tRA was added with AGN193109, the reduction of RARE-luciferase activity was abolished in a dose-dependent manner (Figure 2Bi). Similarly, when mIMCD-3 cells were treated with DEAB, a reduction of basal RARE-luciferase activity was observed, which was reversed when exogenous tRA was added (Figure 2Bii). Note that the RARE-luciferase activity was reversed to a level similar to the vehicle control group at 0.1 nM tRA; the RARE-luciferase activity was saturated by 1 nM tRA, which was about 1.75-fold of that of the vehicle control group and about 3.5-fold of that of the DEAB-treated group (Figure 2Bii). Under culture conditions, when RARE-luciferase transfected mIMCD-3 cells were treated with exogenous tRA alone, the induction of RARE-luciferase activity was weak and did not reach statistical significance (Figure 2Biii), suggesting that the constitutive RARE activity induced by endogenous tRA/RARs was close to saturation for activating the reporter construct.

### Candidate Target Genes of Endogenous tRA/RARs

Two sets of independent biological experiments (N = 3 each) were performed: (i) cells treated with AGN193109, with and without tRA, to identify candidate genes regulated by RARs, and (ii) cells treated with DEAB, with and without tRA, to identify candidate genes regulated by endogenous tRA. Samples taken from these studies were subjected to microarray analysis, in which candidate target genes of endogenous tRA/RARs were defined as those genes regulated in the same direction by both AGN193109 and DEAB, with the regulation partially or completely abolished by exogenous tRA at concentrations used in the earlier studies shown in Figure 2. Partial abolishment included a statistical difference between the “inhibitor-only” and “inhibitor with tRA” groups, or no statistical difference between the “inhibitor with tRA” and “vehicle control” groups.

**Figure 3:**
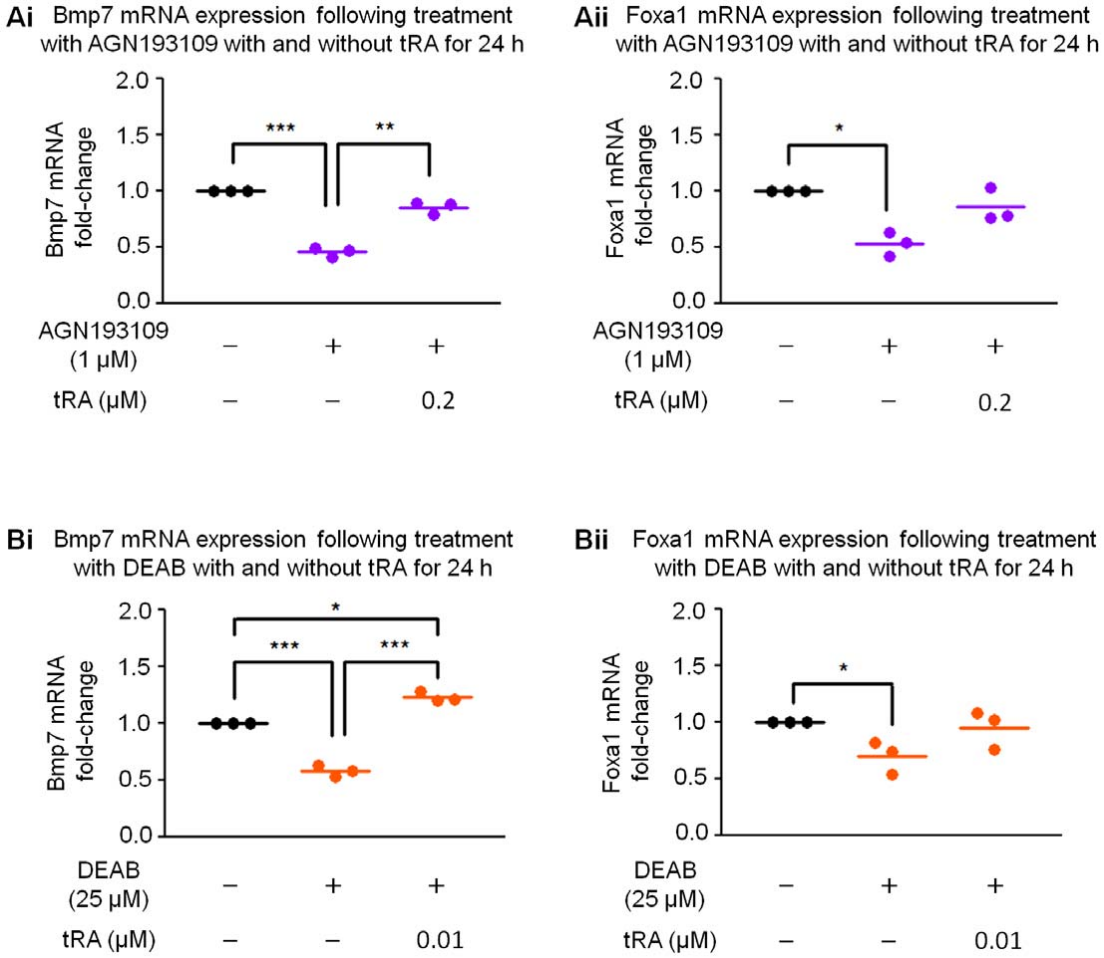
Regulation of Bmp7 and Foxa1 by AGN193109 and 4-(diethylamino)benzaldehyde (DEAB) in pilot study. mRNA expression of Bmp7 (**Ai**) and Foxa1 (**Aii**) was suppressed by AGN193109; the suppression was at least partially abolished in the presence of 0.2 µM tRA. **Bi.** Bmp7 mRNA was suppressed by DEAB; the suppression was reversed to a level slightly higher than basal level in the presence of 0.01 µM tRA. **Bii.** Expression of Foxa1 mRNA was suppressed by DEAB; the suppression was partially abolished in the presence of 0.01 µM tRA. Each dot represents mean value of three technical replicates from a single biological experiment. *, **, and ***: p<0.05, p<0.01, and p<0.001, respectively.

Prior to microarray experiments, a small group of pilot reverse-transcription-quantitative polymerase chain reaction (RT-qPCR) studies was performed to examine the expression of a few genes of interest to verify the effectiveness of our strategy, as well as to guide the process of shortlisting target genes in the ensuing microarray studies. For this purpose, we selected Bmp7, Foxa1, Pax2, and Wnt7b, which have been implicated in kidney development, water transport, and defense/protection mechanism against injury [15], [20], [21], [22], [23]. These genes have been reported in other systems to be regulated by tRA with or without known functional RARE [24], [25], [26], [27]. As shown in Figure 3, Bmp7 and Foxa1 mRNAs were suppressed by both AGN193109 and DEAB; the suppression was at least partially abolished when exogenous tRA was added simultaneously. On the other hand, Wnt7b and Pax2 mRNAs were not significantly regulated by either AGN193109 or by DEAB (data not shown).

**Figure 4:**
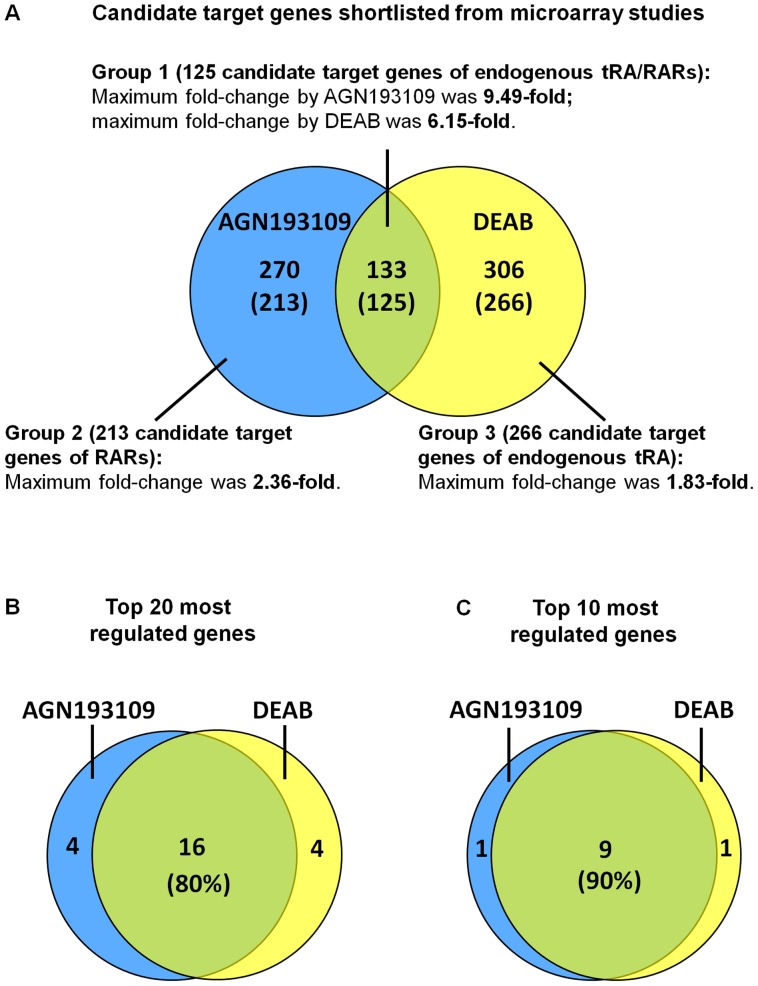
Candidate target genes short-listed from microarray experiments. A. A total of 403 and 439 unique genes were short-listed from AGN193109 experiment and DEAB experiment, respectively. 133 genes were regulated by AGN193109 and DEAB; among them, 125 genes (designated as group 1 genes), were regulated by AGN193109 and DEAB at similar directions, which the regulation was at least partially abolished in the simultaneous presence of exogenous tRA. 270 genes were regulated only by AGN193109, of which regulation of 213 genes (designated as group 2 genes) was at least partially abolished in the simultaneous presence of exogenous tRA; 306 genes were regulated only by DEAB, of which regulation of 266 genes (designated as group 3 genes) was at least partially abolished in the simultaneous presence of exogenous tRA. Note that the fold-changes of intersected genes were higher than those of non-intersected genes. When ranked based on fold-changes, 80% of the top 20 most regulated genes (**B**) and 90% of the top 10 most regulated genes were overlapped (**C**).

Given that the magnitude of regulation for Foxa1 was lower than that of Bmp7, and the effects of exogenous tRA did not reach significance, Foxa1 was used as a cut-off threshold for shortlisting candidate target genes (details described in the Materials and Methods section): 403 and 439 unique genes were generated from AGN193109 experiments and DEAB experiments, respectively. As shown in Figure 4A, there were 133 overlapping genes; of these, 125 were similarly regulated by AGN193109 and by DEAB, and the regulation was at least partially abolished in the simultaneous presence of exogenous tRA. These 125 genes were categorized as the group 1 genes that represent specific target genes of endogenous tRA/RARs. In addition to the group 1 genes, there were 213 genes designated as group 2 genes that were regulated only by AGN193109, but not DEAB. These were candidate genes regulated by RARs, but not endogenous tRA. There were 266 additional genes designated as group 3 genes that were regulated only by DEAB, but not AGN193109. They were considered as candidate genes regulated by endogenous tRA, but independent of RARs (Figure 4A). When the genes were ranked by fold-changes, 16 out of the top 20 and 9 out of the top 10 most highly regulated genes overlapped (Figure 4B and 4C), suggesting that the most regulated genes were dependent on both tRA and RARs. Thus, further analysis was focused on the tRA/RAR-dependent genes (group 1).

**Figure 5:**
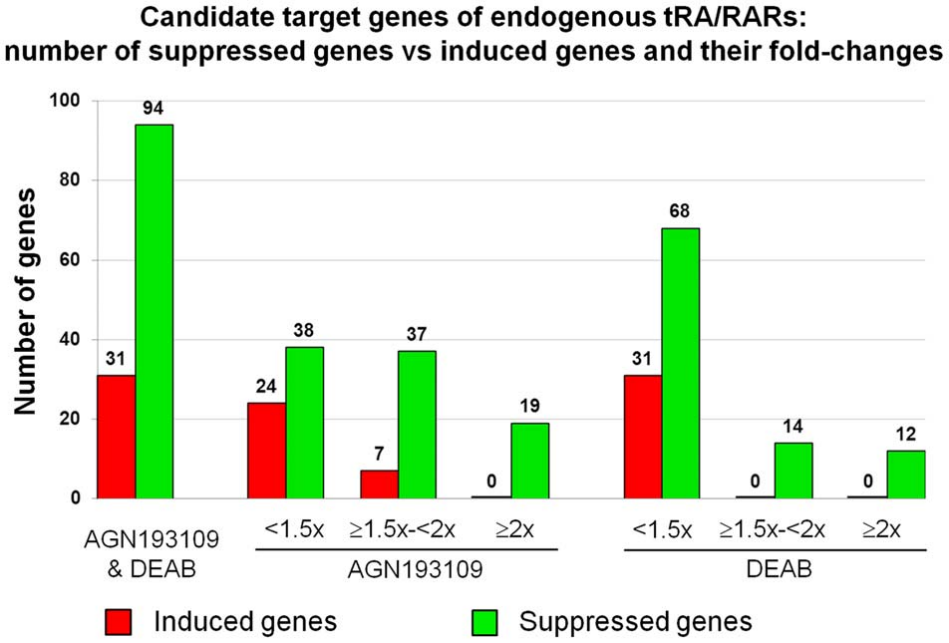
Candidate target genes of endogenous tRA/RARs: number of induced and suppressed genes. A total of 31 and 94 genes were up-(red) and down-(green) regulated, respectively, by AGN193109 and by DEAB; regulation of these genes were at least partially abolished in the presence of tRA. Of the 31 up-regulated genes, 24 were up-regulated by AGN193109 by less than 1.5-fold, 7 by more than 1.5-fold but less than 2-fold, and none by 2-fold and more; none of the genes were up-regulated by DEAB by 1.5-fold and more. Of the 94 down-regulated genes, 38 were down-regulated by AGN193109 by less than 1.5-fold, 37 by more than 1.5-fold but less than 2-fold, and 19 by 2-fold and more; 68 were down-regulated by DEAB by less than 1.5-fold, 14 by more than 1.5-fold but less than 2-fold, and 12 by 2-fold and more.

The number and fold-changes of all group 1 genes are summarized in Figure 5. The total number of genes suppressed by both AGN193109 and DEAB were approximately 3-fold more than those induced by both AGN193109 and DEAB. Of note, there were 19 and 12 genes suppressed by AGN193109 and DEAB by 2-fold or more, respectively, but none was induced by 2-fold or more, indicating that tRA/RARs play a more important role in maintaining, rather than suppressing, gene expression. The top 20 most down- and up-regulated genes within group 1 are listed in Table 1 and Table 2, respectively. The complete lists of genes regulated by AGN193109 and by DEAB, genes regulated only by AGN193109 and genes regulated only by DEAB, are compiled in Tables S1, S2, S3. Original raw data of microarray experiments have been deposited in NCBI’s Gene Expression Omnibus (GEO) and are accessible through GEO Series accession number GSE33955 (http://www.ncbi.nlm.nih.gov/geo/query/acc. cgi?acc = GSE33955).

**Table 1:** Top 20 most down-regulated genes.

	Gene Symbol	AGN193109	AGN193109+ tRA	DEAB	DEAB + tRA	Entrez Genes
1.	Ppbp	−9.49	−1.53	−6.15	+1.85	57349
2.	Dhrs3	−8.59	−3.78	−5.02	+2.03	20148
3.	Sprr1a	−6.85	−1.38	−4.62	+2.53	20753
4.	Cpm	−5.15	−1.69	−2.30	+1.33	70574
5.	9930023K05Rik	−4.36	−1.59	−2.35	+1.60	226245
6.	Tns1	−3.22	−1.82	−2.29	+1.27	21961
7.	Itga2	−3.22	−1.16	−2.56	+1.25	16398
8.	Klhdc7a	−3.10	−1.87	−2.11	+1.03	242721
9.	Csn3	−2.91	−1.91	−2.18	+1.18	12994
10.	Sorcs2	−2.66	−1.46	−2.05	+1.18	81840
11.	Ebf1	−2.66	−1.43	−2.43	+1.33	13591
12.	Lcn2	−2.48	−1.28	−1.78	+1.16	16819
13.	2310007B03Rik	−2.31	−1.58	−1.42	1.00	71874
14.	Galns	−2.27	−1.55	−1.69	+1.07	50917
15.	Npr3	−2.21	−1.03	−1.99	+1.52	18162
16.	Muc20	−2.09	−1.84	−2.18	+1.08	224116
17.	Clca4	−2.07	+1.31	−1.78	+1.94	229927
18.	Upk3b	−2.05	−1.50	−1.47	+1.07	100647
19.	Slc37a1	−2.00	−1.33	−1.53	+1.16	224674
20.	Hrsp12	−1.98	−1.27	−1.65	+1.06	15473

Shown here are mean fold-changes of gene expression compared to vehicle control from three experimental groups. Genes were sorted by fold-changes of AGN193109 group compared to vehicle group. Minus and plus numbers indicate folds of suppression and induction, respectively, in comparison to the vehicle control group, which was normalized as 1.

**Table 2:** Top 20 most up-regulated genes.

	Gene Symbol	AGN193109	AGN193109+tRA	DEAB	DEAB+tRA	Entrez Gene
1.	Anxa8	+1.73	−1.10	+1.43	−1.54	11752
2.	Ptgs2	+1.67	+1.48	+1.24	−1.23	19225
3.	Gsdma	+1.61	−1.01	+1.39	−1.33	57911
4.	Cpeb2	+1.57	+1.18	+1.24	−1.15	231207
5.	Ahnak2	+1.57	−1.11	+1.42	−1.42	100041194
6.	Casp14	+1.56	−1.11	+1.16	−1.31	12365
7.	Peg10	+1.52	+1.21	+1.35	−1.16	170676
8.	Ndrg1	+1.45	−1.02	+1.32	−1.24	17988
9.	Rnf39	+1.45	+1.11	+1.18	−1.19	386454
10.	Pkp1	+1.40	+1.11	+1.25	−1.20	18772
11.	Alcam	+1.39	1.00	+1.36	−1.05	11658
12.	Timp3	+1.39	−1.03	+1.12	−1.15	21859
13.	Adora1	+1.38	+1.15	+1.27	−1.08	11539
14.	Car5b	+1.36	+1.03	+1.13	−1.28	56078
15.	Atp6v0a4///D630045J12Rik	+1.34	+1.11	+1.21	−1.23	140494///330286
16.	Egr1	+1.33	+1.24	+1.07	−1.02	13653
17.	Ly6c1///Ly6c2	+1.31	−1.01	+1.14	−1.20	17067///100041546
18.	Cav1	+1.30	+1.02	+1.20	−1.11	12389
19.	Lama3	+1.28	−1.02	+1.04	−1.21	16774
20.	Lmna	+1.26	−1.02	+1.17	−1.17	16905

Shown here are mean fold-changes of gene expression compared to vehicle control from three experimental groups. Genes were sorted by fold-changes of AGN193109 group compared to vehicle group. Minus and plus numbers indicate folds of suppression and induction, respectively, in comparison to the vehicle control group, which was normalized as 1.

### Validation of Microarray Results

The top 20 most highly regulated genes shortlisted from the microarray experiments were all down-regulated by AGN193109 and by DEAB. Regulation of these genes was validated by RT-qPCR. As shown in Table 3, among the 20 genes examined, 19 were significantly down-regulated by AGN193109; suppression of all these genes, except Muc20, was at least partially abolished in the presence of exogenous tRA. The same 19 genes were also significantly down-regulated by DEAB and suppression was abolished in the presence of exogenous tRA. Thus, while Csn3, which was not amplified, and Muc20 need further investigation, the remaining 18 genes, along with Bmp7 and Foxa1 examined in the pilot study, were confirmed as specific target genes of endogenous tRA/RARs.

**Table 3:** Validation of top 20 most down-regulated genes.

	Gene Symbol	AGN193109	AGN193109+tRA	DEAB	DEAB+tRA
1.	Dhrs3	0.01±0.0009***	0.14±0.0495∧∧∧ **	0.04±0.0052***	2.88±0.4050+++ **
2.	Sprr1a	0.05±0.0126***	0.60±0.0575∧∧∧	0.15±0.0195***	4.11±0.3857+++ ***
3.	Ppbp	0.07±0.0056***	0.73±0.0586∧∧∧ *	0.14±0.0129***	2.56±0.1259+++ ***
4.	9930023K05Rik	0.12±0.0072***	0.63±0.0326∧∧∧ **	0.28±0.0270***	2.09±0.1978+++ **
5.	Cpm	0.13±0.0217***	0.47±0.0053∧∧ *	0.31±0.0240***	1.48±0.0393+++ **
6.	Tns1	0.14±0.0282**	0.37±0.0587∧ *	0.32±0.0272***	1.52±0.0506+++ *
7.	Lcn2	0.17±0.0090***	0.70±0.0604∧∧∧ *	0.26±0.0057***	1.53±0.1460+++ *
8.	Itga2	0.19±0.0360**	0.90±0.1579∧∧	0.27±0.0064***	1.44±0.1289+++ *
9.	Npr3	0.21±0.0356**	0.93±0.1878∧∧	0.35±0.0374**	2.09±0.1123+++ **
10.	Klhdc7a	0.21±0.0110**	0.43±0.0740∧ *	0.34±0.0249***	1.27±0.0792+++
11.	Sorcs2	0.25±0.0625**	0.68±0.0667∧	0.34±0.0026***	1.34±0.1383+++
12.	Clca4	0.26±0.0526***	1.70±0.3294∧∧∧	0.53±0.0504**	2.69±0.1611+++ **
13.	2310007B03Rik	0.25±0.0269***	0.55±0.0100∧∧ **	0.59±0.0356**	1.12±0.0751++
14.	Muc20	0.31±0.0248***	0.41±0.0573**	0.36±0.0440**	1.21±0.0852++
15.	Upk3b	0.35±0.0061***	0.65±0.0077∧∧∧	0.58±0.0125***	1.16±0.0190+++ **
16.	Galns	0.37±0.0007***	0.69±0.0407∧∧	0.44±0.0507**	1.16±0.1101+++
17.	Slc37a1	0.40±0.0781**	0.76±0.0342∧	0.61±0.0181***	1.30±0.0573+++ **
18.	Ebf1	0.44±0.0681**	0.80±0.0313∧	0.54±0.0237**	1.55±0.1321+++ **
19.	Hrsp12	0.52±0.0435**	0.80±0.0561∧∧	0.57±0.0382**	1.19±0.0826+++
20.	Csn3	NA	NA	NA	NA

Shown here are means of gene expression levels relative to vehicle control group ± standard errors (SE); the vehicle control group was normalized as 1. Taking Dhrs3 expression as an example, 0.01 and 2.88 in the AGN193109 group and the DEAB+tRA group indicate 100-fold reduction and 2.88-fold induction relative to the vehicle group, respectively.

* p<0.05 vs vehicle,

** p<0.01 vs vehicle,

*** p<0.001 vs vehicle;

∧ p<0.05 vs AGN193109,

∧∧ p<0.01 vs AGN193109,

∧∧∧ p<0.001 vs AGN193109;

+ p<0.05 vs DEAB,

++ p<0.01 vs DEAB,

+++ p<0.001 vs DEAB; NA: Not amplified.

### Gene Ontology Analysis

**Figure 6:**
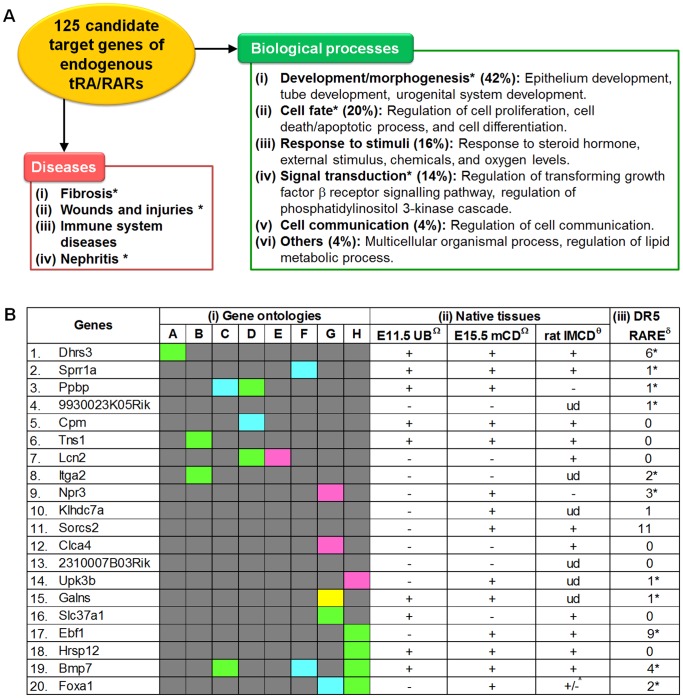
Gene ontologies, expression in native tissues, and presence of retinoic acid response element (RARE). A. Enrichment analysis on the 125 candidate target genes showed that they are associated with many biological processes (green box) and diseases (red box), some of which are highly relevant to renal physiology and pathology. Those marked with an asterisk were reported to be associated with tRA/RAR signaling. The full list of gene ontologies for the 125 candidate target genes are in Table S4. **B.** (**i**) The 20 validated target genes of endogenous tRA/RARs were reported to be involved in retinol metabolism (A), cell-cell, cell-substrate interaction (B), ureteric bud branching (C), immune/inflammatory processes (D), oxidative stress (E), repair/regeneration (F), ion/solute/water transport and metabolism (G), and gene transcription/translation (H); functions of 9930023K05Rik, Klhdc7a, Sorcs2, and 2310007B03Rik are not yet known. Pink: GeneGO Metacore™, Yellow: DAVID [50], Green: GeneGO Metacore™ and DAVID, Blue: additional literature review [20], [22], [45], [48], [51], [52], [53]. The full list of gene ontologies for the 20 validate target genes are in Table S5. (**ii**) Expression of the validated genes in native samples, available through Gene Expression Omnibus datasets (Ω: GSE6290, θ: GSE7891), was reviewed. E11.5 UB: embryonic day 11.5 ureteric bud cells from mice (three samples), E15.5 mCD: embryonic day 15.5 medullary collecting duct cells from mice (three samples), rat IMCD: inner medullary collecting duct cells from rat (one sample from 6-week-old and two samples from 10-week-old). +: present, −: absent, ud: undetermined; ?: present in 6-week-old sample but absent from 10-week-old samples. (**iii**) The number of direct-repeat 5 (DR5) RARE that are present in the validated genes in whole mouse genome is summarized here, based on report from Lalevee et al. [4]. *: At least one of the DR5 RAREs is present within 10 kb from transcriptional start site and from gene end.

The 125 candidate target genes of endogenous tRA/RARs were subjected to GeneGO Metacore™ analysis to determine if they are enriched for a particular set of biological processes and diseases. As shown in Table S4, the top 50 biological processes in which the candidate target genes are involved can be broadly categorized into six main groups: 42% of the top 50 biological processes are under the umbrella of developmental processes or morphogenesis, 20% are in cell fate determination, 16% are in response towards stimuli, 14% are in regulation of signal transduction, 4% are in cell communication, and the remaining 4% are in other processes (Figure 6A). These genes were also reported to be dysregulated in some diseases, including fibrosis, wounds healing, immune system diseases, and renal inflammation (Figure 6A).

Expression of the validated genes in native tissues was determined using two GEO series deemed relevant to mIMCD-3 cells: (i) E11.5 mouse UBs and E15.5 mouse medullary CD cells (GEO accession: GSE6290), contributed by GenitoUrinary Development Molecular Anatomy Project (GUDMAP) (http://www.gudmap.org/), and (ii) inner medullary CD cells derived from 6- and 10-week-old rat kidney (GEO accession: GSE7891), contributed by Uawithya et al [28]. There were three biological replicates in each of the database; a gene was considered as positively expressed if at least two out of three replicates had positive hybridization signal. As shown in Figure 6B, expression of all the 20 genes, except for 9930023K05Rik, 2310007B03Rik, and Itga2, was found in at least one native sample of UB/CD cells. In addition, by referring to a recently published database of DR5 RARE in mouse whole genome [4], it was found that 13 out of the 20 validated genes contain at least one DR5 RARE. Of these 13 genes, 11 of them have at least one DR5 RARE within 10 kb from the transcription start sites and from gene ends (Figure 6B), which are regions deemed highly relevant for transcriptional activity modulation [4].

Gene ontologies for each validated gene were also examined and classified since these genes showed the greatest amount of change (Figure 6B). These genes were reported to be involved in regulating the conversion of tRal into tRol (Dhrs3), regulating UB branching and kidney development (Ppbp and Bmp7), maintaining CD cell polarity and integrity by regulating cell-cell and cell-substrate interactions (Tns1 and Itga2), regulating cellular responses to stress and modulating tissue repair (Sprr1a, Cpm, Lcn2, and Bmp7), regulating water and solute transport mechanisms, and kidney stone formation (Npr3, Foxa1, Clca4, Slc37a1, and Galns). The known functions of these identified tRA/RAR target genes might help in understanding how tRA/RARs function at the molecular level in the UB/CD cell lineage and guide future studies. In addition, further studies of the function of some genes with unknown functions (9930023K05Rik, Klhdc7a, Sorcs2, and 2310007B03Rik) or functions not yet associated with UB/CD cells and the kidney (Ebf1, Upk3b, and Hrsp12) might not only help to better understand the functions of these genes, but also might help to discover novel functions of the UB/CD cell lineage. The complete list of gene ontologies of the validated genes was listed in Table S5.

## Discussion

In this study, we have detected the presence of constitutive RAR activation following continuous stimulation by endogenous tRA in mIMCD-3 cells (Figure 2). Specifically, AGN193109 and DEAB treatment reduced RARE-luciferase activity, indicating the presence of basal tRA/RAR signaling in mIMCD-3 cells; this effect was abolished by exogenous tRA, in a dose-dependent manner, confirming the specificity of AGN193109 and DEAB. Interestingly, exogenous tRA treatment alone resulted in only weak induction of RARE-luciferase activity, although induction was 1.75-fold higher compared with the vehicle control group when exogenous tRA and DEAB were added simultaneously to the cells. We interpreted this to mean that there was already constitutive RAR activation by endogenous tRA in mIMCD-3 cells and that further activation was limited by low cellular uptake of exogenous tRA, availability of RARs and/or transcriptional co-regulators. On the other hand, when endogenous tRA synthesis was abolished by DEAB, enhanced cellular uptake of exogenous tRA and/or enhanced expression of RARs and co-factors might have taken place, thus potentiating RARE-luciferase induction to significantly higher than the basal level (Figure 2 Bii). Thus, for cells with constitutively active endogenous tRA/RAR signaling such as mIMCD-3 cells, instead of treating them directly with exogenous tRA or RAR agonists to identify tRA/RAR target genes, it would be more appropriate to determine genes differentially regulated when endogenous tRA/RAR signaling was inhibited (Figure 1).

By treating mIMCD-3 cells with AGN193109 and DEAB, with and without exogenous tRA, we were able to catalogue a list of candidate target genes specifically regulated by both endogenous tRA and RARs. Note that when all shortlisted genes were overlapped directly the intersected genes appeared to be the minority (Figure 4A). However, only when the top 20 and top 10 most highly regulated genes were overlapped, did the intersected genes become the majority (Figure 4B and 4C). This finding was also reflected by the greater fold-changes observed for the intersected genes compared with the non-intersected genes (Figure 4A). Thus, the data suggest that RAR-independent tRA signaling or tRA-independent RAR signaling might regulate specific gene subsets, respectively. Nonetheless, genes most dependent on retinoid signaling in mIMCD-3 cells were those regulated by the canonical tRA/RAR signaling. We also found that endogenous tRA/RAR signaling plays a primary role as “inducer” to support gene expression, rather than as “suppressor” to inhibit gene expression in mIMCD-3 cells, since genes suppressed by AGN193109 and by DEAB greatly exceeded genes induced by AGN193109 and by DEAB, in number of genes and magnitude of regulation (Figure 5).

Among the 20 validated target genes of endogenous tRA/RARs, including Foxa1 and Bmp7 identified in the pilot study (Figure 6B), some were reported to be regulated by tRA and/or RARs in previous reports, i.e., Dhrs3 (up-regulated) [29], [30], Sprr1a (down-regulated) [31], [32], Cpm (down-regulated) [33], Itga2 (up-regulated) [34], Ebf1 (up-regulated) [35], Lcn2 (up-regulated) [36], Bmp7 (up-regulated) [24], and Foxa1 (up-regulated) [25]. The remaining genes, i.e., Ppbp, 9930023K05Rik, Tns1, Klhdc7a, Sorcs2, 2310007B03Rik, Galns, Npr3, Clca4, Upk3b, Slc37a1, and Hrsp12, represent novel target genes of endogenous tRA/RARs, at least in mIMCD-3 cells. Except for 9930023K05Rik, 2310007B03Rik, and Itga2, all validated genes are expressed in native samples of UB/CD cells, suggesting that they are of physiological relevance. We also noted that half of these genes contain at least one DR5 RARE; among them, almost all have at least one DR5 RARE within 10 kb from transcription start sites and from gene ends, which are the regions deemed most relevant for transcriptional activity modulation [4]. These genes might have been regulated via their DR5 RARE(s), although other mechanisms, e.g., through other forms of RARE or though RARE-independent mechanisms, might also be involved. It is also worth noting that several well-established target genes of tRA that have more than one functional DR5 RAREs, e.g., Rarb and Cyp26a1 [25], were not shortlisted in the microarray experiments. In fact, there was no statistically significant difference in the retinoid binding proteins, metabolizing enzymes, and the retinoid nuclear receptors, between the vehicle, inhibitors alone, and inhibitors with exogenous tRA groups, as shown in the microarray data. This could be explained by the cell type-dependent activity of tRA, but treatment length might also have an impact. Additional studies at earlier and later time points should complement the existing profile of endogenous tRA/RAR target genes in mIMCD-3 cells.

Among the top 50 biological processes that were significantly enriched (Figure 6A), 42% was within the category of development and morphogenesis, in agreement to the established role for tRA as a morphogen. Apart from kidney development and other previously reported tRA/RAR-dependent biological processes [7], [37], these genes are also involved in responses to external stimuli, including oxygen levels, hormones, and chemicals, consistent with the functions of CD cells to withstand the highly variable oxygen levels, particularly in the medulla, as well as in regulating water and solute transport.

Besides the classical functions of CD cells, emerging publications are suggesting novel functions for these cells, e.g., in regulating inflammation, epithelial-mesenchymal transition and fibrogenesis [38], [39], as well as in defense against bacterial infection [40], etc. Moreover, post-natal vitamin A deficiency has been associated with renal anomalies, including increased incidence of pyelonephritis, kidney inflammation and fibrosis, polyuria and dysregulated urinary ion/solute content, urolithiasis, and delayed tissue repair [7], [41], [42], [43], [44]. Thus, the roles for the tRA/RAR target genes identified in this study in the aforementioned renal anomalies associated to post-natal vitamin A deficiency deserve further studies.

Among the validated target genes, the top three genes that were most dramatically regulated by tRA/RARs could have important roles in mediating the biological processes and diseases mentioned above. For instance, Ppbp could be regulating kidney development [45], inflammation [46], and defense against bacterial infection [47]; Sprr1a is another gene highly dependent on tRA/RAR signaling in mediating tissue repair and regeneration [48] relevant to kidney injury. It is important that while some validated genes, e.g., Dhrs3, Cpm, and Tns1, are expressed as cellular components, other genes, e.g., Ppbp and Lcn2, are expressed as secreted proteins. This supports the view that tRA/RARs not only regulate CD cell function in an autocrine manner, but also modulate the function and health of the kidney or the whole body in a non-autocrine manner. The importance of tRA/RAR signaling in CD cells is also supported by the regulation of Dhrs3 by AGN193109, DEAB and exogenous tRA. Dhrs3 is involved in converting retinaldehyde back to vitamin A to reduce tRA biosynthesis [30]. The pronounced regulation of Dhrs3 might represent a self-regulatory mechanism of endogenous tRA/RAR signaling in CD cells.

In summary, we have detected endogenous tRA/RAR activity in mIMCD-3 cells, an *in vitro* cell model of the UB/CD cell lineage. Using this model, a panel of genes regulated by both endogenous tRA and RARs has been identified. Many of these genes represent novel target genes of endogenous tRA/RARs. We propose that endogenous tRA/RARs may play crucial roles in kidney development and in maintaining normal function of CD cells and the kidney, at least in part, by regulating these tRA/RAR target genes. Given the complexity of retinoid signaling, which is highly dependent on cell type and environment, further studies are warranted to examine the regulation of these genes by tRA/RAR signaling *in vivo* and to explore the potential role of these endogenous tRA/RAR target genes in normal and abnormal renal function.

## Materials and Methods

### Cell Culture

mIMCD-3 cells (LGC Standards, Middlesex, UK) were routinely grown in DMEM-F12 (PAA Laboratories Ltd, Somerset, UK) containing penicillin (100 IU/ml), streptomycin (100 µg/ml) (PAA Laboratories Ltd) and amphotericin B (2.5 µg/ml) (Life Technologies Ltd, Paisley, UK) herein referred as complete medium, supplemented with 5% fetal calf serum (FCS; Life Technologies Ltd), at 37°C and 5% CO_2_.

### Immunocytochemistry

Cells were seeded at 6×10^5^ cells/35 mm^2^ dish in 2 ml complete medium, supplemented with 5% FBS. After an overnight culture until confluent, cells were fixed with 5% ice-cold formalin for 15 min on ice, followed by washing with three changes of PBS, 5 min each wash. Cells were then permeabilized with 0.1% Triton X-100 for 3 min and washed with three changes of PBS, 5 min each wash. After fixation and permeabilization, cells were incubated with 1% BSA for 2 h at room temperature to reduce non-specific antibody binding, followed by 1 h incubation of rabbit anti-AQP2 IgG (dilution 1∶150, Millipore, Watford, UK), or mouse anti-E-cad IgG2a (dilution 1∶50, BD Biosciences, Oxford, UK), at room temperature. Cells incubated with non-immune rabbit IgG and non-immune mouse IgG2a (Insight Biotechnology Ltd, Middlesex, UK) at the same concentrations served as negative controls for anti-AQP2 IgG and for anti-E-cad IgG2a, respectively. Cells were then washed with three changes of PBS, 5 min each wash, and were incubated with goat anti-rabbit conjugated with Alexa Fluor 488 (dilution 1∶1000, Life Technologies Ltd) for AQP2 detection or goat anti-mouse conjugated with Alexa Fluor 555 (dilution 1∶1000, Life Technologies Ltd) for E-cad detection, for 1 h at room temperature. At the end of secondary antibody incubation, cells were washed with three changes of PBS, 5 min each wash. One milliliter of fresh PBS was then pipetted onto the cell monolayer and fluorescence microscopy was performed immediately.

### Microscopy

Phase-contrast and fluorescence microscopy was performed on a Nikon Eclipse TE2000-S epifluorescence microscope equipped with a standard RGB filter wheel (Nikon Instruments Europe B.V., Amstelveen, The Netherlands). Images were captured with a DXM1200F Nikon digital camera (Nikon UK Limited, Surrey, UK), then processed and merged with Adobe Photoshop (Adobe Systems Europe Ltd, Uxbridge, UK).

### Reagents

tRA (Sigma-Aldrich Company Ltd, Dorset, UK) was reconstituted in 100% ethanol to 10 mM; DEAB [19] (Sigma-Aldrich Company Ltd) was reconstituted in 100% dimethylsulphoxide (DMSO) (Sigma-Aldrich Company Ltd) to 25 mM; AGN193109 [18], was reconstituted in 100% DMSO to 1 mM. All reagents were first diluted with their respective diluents to 1000× more concentrated than the working concentration, then diluted 1000× with culture medium to the working concentrations immediately before treatment was commenced. Control groups were treated with 0.1% ethanol and/or 0.1% DMSO.

### Transient Transfection

Lipofectamine™ LTX, Plus™ reagents and Opti-MEM® I Reduced Serum Medium (Life Technologies Ltd) were used in transient transfection of plasmid DNA according to the manufacturer’s instructions. pmaxGFP (Lonza Wokingham Ltd, Berkshire, UK) was used to assess transfection efficiency; the transfection efficiency was estimated to be around 70% (Figure S1). pGL3-RARE-luciferase plasmid [49], was used to assess RAR-dependent endogenous tRA activity. pCI-β-galactosidase plasmid (Promega, Madison, USA) was used for normalization of RARE-luciferase activity. Background signal was determined from wells treated with transfection medium only without plasmid DNA. Ratio of pGL3-RARE-luciferase: pCI-β galactosidase was 5∶1 when co-transfected into mIMCD-3 cells. Cells were lysed with Reporter Lysis Buffer (Promega UK, Southampton, UK); luminescence signal was detected with Luciferase Assay System and Beta-Glo® Assay System (Promega UK) and was expressed as relative light unit (RLU). RLU of treatment group was normalized to vehicle control group and expressed as fold-change.

### RNA Extraction and Reverse Transcription-quantitative Polymerase Chain Reaction (RT-qPCR)

Total RNA was extracted using RNeasy® Mini Kit and QIAshredder spin column (Qiagen Ltd, West Sussex, UK) following manufacturer’s protocol. Quantity and quality of total RNA was determined with a NanoDrop® ND-1000 machine (Labtech International Ltd, East Sussex, UK); an absorbance ratio of 260/280 and 260/230 for all total RNA samples were within the range of 1.8–2.0. Reverse transcription was performed using Oligo-d(T) primer and Omniscript® Reverse Transcription Kit (Qiagen Ltd) following manufacturer’s protocol. qPCR was performed using Taqman® universal master mix and Taqman® Gene Expression Assays (Life Technologies Ltd), as listed in Table 4. Conditions for qPCR reactions were as follows: 50°C for 2 min, 95°C for 10 min, 40 cycles of 95°C for 15 s and 60°C for 1 min. Gene expression was computed using the 2−^ΔΔ^CT formula with glyceraldehyde 3-phosphate dehydrogenase (Gapdh) as an internal control; gene expression of the vehicle control group was normalized as 1. Expression of Gapdh was found to be stable across all experimental groups (Figure S2).

**Table 4:** Assay ID and amplicon length of Taqman® Gene Expression Assays used.

	Gene	Assay ID	Amplicon length
1.	Bmp7	Mm00432101_m1	82
2.	Clca4	Mm00519742_m1	68
3.	Cpm	Mm01250796_m1	63
4.	Csn3	Mm02581554_m1	99
5.	Dhrs3	Mm00488080_m1	84
6.	Ebf1	Mm00432948_m1	59
7.	Foxa1	Mm00484713 _m1	68
8.	Galns	Mm00489576_m1	80
9.	Gapdh	Mm99999915 _m1	107
10.	Hrsp12	Mm00476177_m1	73
11.	Itga2	Mm00434371_m1	63
12.	Klhdc7a	Mm00557861_s1	117
13.	Lcn2	Mm01324470_m1	84
14.	Muc20	Mm00524818_m1	65
15.	Npr3	Mm00435329_m1	63
16.	Pax2	Mm01217939_ m1	55
17.	Ppbp	Mm00470163_m1	62
18.	Slc37a1	Mm00461949_m1	78
19.	Sorcs2	Mm00473050_m1	64
20.	Sprr1a	Mm01962902_s1	94
21.	Tns1	Mm00452886_m1	91
22.	Upk3b	Mm00558406_m1	89
23.	Wnt7b	Mm00437357_ m1	88
24.	9930023K05Rik	Mm00554061_m1	110
25.	2310007B03Rik	Mm00549644_m1	73

### Microarray

Samples were prepared using Ambion® WT kit (Life Technologies Ltd), Affymetrix fragmentation and labeling kit, and Affymetrix wash, stain, and hybridization kit (Affymetrix UK Ltd, High Wycombe, UK). Mouse Gene 1.0 ST array (Affymetrix UK Ltd) was used for gene expression profiling. Agilent Nano Chip and bioanalyser (Agilent Technologies UK Ltd, Edinburgh, UK) were used to assess the quality of total RNA; there was no evident of RNA degradation, as shown in Figure S3. Nucleotide size of sense-cDNA was also examined before and after fragmentation. An Affymetrix® Gene Control Console software was used for data normalization using the Robust Multichip Average method. Candidate target genes were short-listed using an Qlucore Omics Explorer software with multiple comparison analysis, by using Foxa1 as the cut-off threshold of statistical significance (variance = 0.098, q = 0.65 for AGN193109 experiment; variance = 0.053, q = 0.30 for DEAB experiment). The RNA samples used for microarray experiments were subsequently used in the validation experiments.

### Gene Ontology Analysis

Gene ontology analysis was performed using Database for Annotation, Visualization and Integrated Discovery (DAVID) (http://david.abcc.ncifcrf.gov/) [50] and GeneGO Metacore™ (GeneGO Inc., St. Joseph, Michigan); additional literature reviews were performed to complement gene ontology analysis.

### Experimental Protocols

For reporter assays involving AGN193109 and tRA, mIMCD-3 cells were seeded into a 24-well plate at 4×10^4^ cells/well in 500 µl of DMEM-F12 medium without antibiotics and anti-mycotic, supplemented with 5% FCS. After culturing for 16–24 h to about 70% confluence, cells were co-transfected with pGL3-RARE-luciferase and pCI-β-galactosidase plasmids, and cultured for another 5 h. Medium was then changed to fresh complete medium supplemented with 1% FCS. After an overnight incubation, medium was changed to fresh complete medium supplemented with 1% FCS containing 1 µM AGN193109, with and without 0.01 µM, 0.1 µM, or 1 µM tRA, and cultured for 24 h. For reporter assays involving tRA alone, the protocol was similar as above, except that cells were treated with 0.001 µM, 0.01 µM, or 0.1 µM tRA for 24 h.

For reporter assays involving DEAB and tRA, mIMCD-3 cells were seeded into a 24-well plate at 2×104 cells/well in 500 µl of DMEM-F12 medium without antibiotics and anti-mycotic, supplemented with 5% FCS. Cells were cultured for 16–24 h to about 40–50% confluence, and medium was changed to fresh DMEM-F12 without antibiotics and anti-mycotic, supplemented with 1% FCS, and contained 25 µM DEAB or vehicle. Cells were then cultured for 16–24 h until about 70% confluence, after which medium changing was repeated as described above and cells were co-transfected with pGL3-RARE-luciferase and pCI-β-galactosidase plasmids. After an overnight incubation, medium was again changed to fresh DMEM-F12 without antibiotics and anti-mycotic, supplemented with 1% FCS, and contained 25 µM DEAB, with and without 0.0001 µM, 0.001 µM, 0.01 µM, or 0.1 µM tRA. Cells were then cultured for 24 h.

For RT-qPCR and microarray experiments, cells were seeded at 1.2–2×105 cells/35 mm^2^ dish in 2 ml complete medium, supplemented with 5% FCS. After an overnight culture, medium was changed to fresh complete medium supplemented with 1% FCS. After an overnight culture, medium was again changed to fresh complete medium supplemented with 1% FCS, containing 1 µM AGN193109 with and without 0.2 µM tRA, or 25 µM DEAB with and without 0.01 µM tRA, and cultured for 24 h.

Control groups were treated with vehicle in all aforementioned protocols. For reporter assays, three independent experiments, with triplicate or quadruplicate wells for each group, were performed. For microarray and RT-qPCR experiments, three independent experiments were performed.

### Statistical Test for Reporter Assay and qPCR

For reporter assays and RT-qPCR experiments, One-way Analysis of Variance (ANOVA) statistical analysis with Tukey post-test was performed on log-transformed fold-change values using GraphPad Prism, Version 4.0. p<0.05 was considered as statistically significant.

## Supporting Information

Figure S1 **Transfection efficiency assessed from green fluorescent protein (GFP) expression.** Around 60% to 70% of the total mIMCD-3 cells transfected with pmaxGFP plasmid expressed GFP (green). No GFP expression was observed in cells where only transfection reagents were added. Original magnification was 100×.
(TIFF)

Figure S2 **Amplification plot of glyceraldehyde 3-phosphate dehydrogenase (Gapdh).** Expression of Gapdh did not vary much between the vehicle control group and 24 h treatment of AGN193109 with and without tRA (**A**), and of DEAB with and without tRA (**B**), evident by a tight overlapping amplification plots across all the samples. Representative amplification plots derived from technical triplicates of a single biological experiment are shown here.
(TIFF)

Figure S3 **RNA integrity of samples for microarray experiments.** Integrity of RNA samples from AGN193109 experiment (**A**) and DEAB experiment (**B**) was examined with bioanalyser. The two sharp major peaks correspond to 18s and 28s ribosomal RNA, respectively. The high RNA Integrity Number (RIN) values, within the range of 9.70–10.00, suggest a good quality of RNA samples with minimum degradation of RNA. There is no evidence of genomic DNA contamination in the RNA samples, given the presence of thin and sharp 18s and 28s RNA peaks, as well as absence of additional peaks other than the expected ribosomal RNA peaks.
(TIFF)

Table S1 133 intersected genes.
(XLS)

Table S2 270 genes regulated by AGN193109.
(XLS)

Table S3 306 genes regulated by DEAB.
(XLS)

Table S4 Gene ontology for 125 candidate target genes.
(XLS)

Table S5 Gene ontology for validated genes.
(XLS)
