# Supplementary figures and images for: Retinoic Acid Receptor-Dependent, Cell-Autonomous, Endogenous Retinoic Acid Signaling and Its Target Genes in Mouse Collecting Duct Cells
Source: PLoS One. 2012 Sep 26;7(9):e45725. doi: 10.1371/journal.pone.0045725 (PMC3458940; doi:10.1371/journal.pone.0045725)

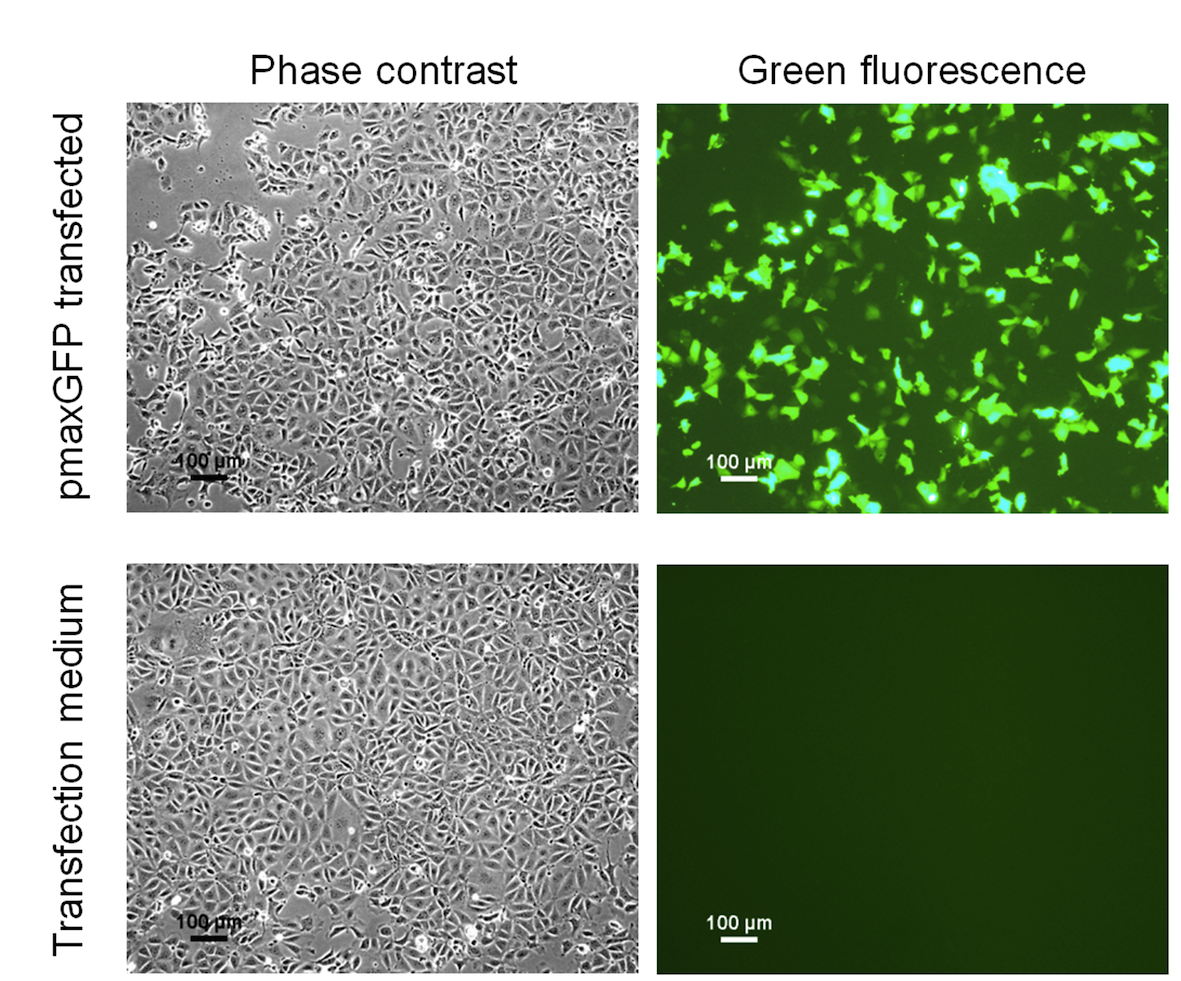

Supplement: Figure S1 — Transfection efficiency assessed from green fluorescent protein (GFP) expression. Around 60% to 70% of the total mIMCD-3 cells transfected with pmaxGFP plasmid expressed GFP (green). No GFP expression was observed in cells where only transfection reagents were added. Original magnification was 100×. (TIFF) [file pone.0045725.s001.tiff]

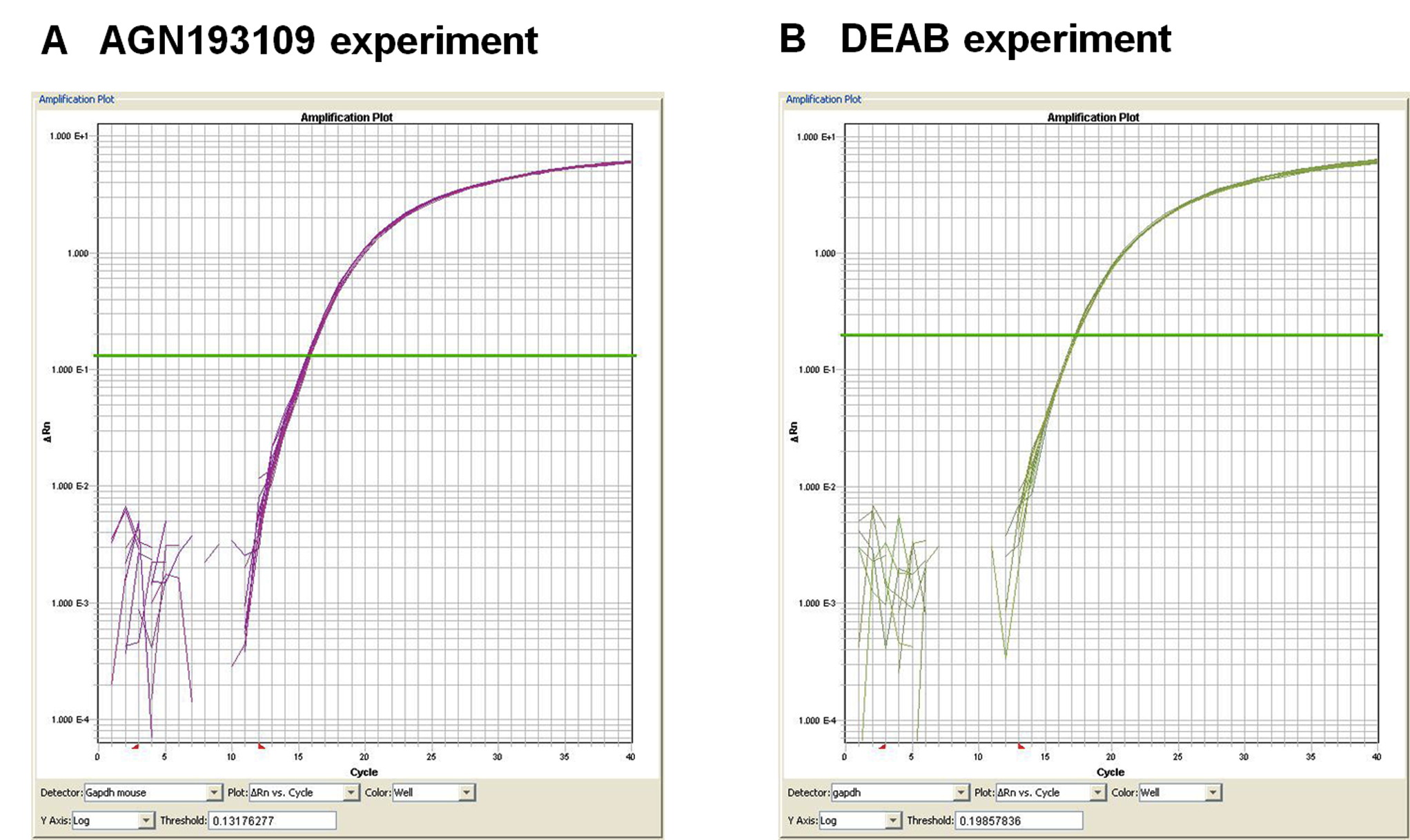

Supplement: Figure S2 — Amplification plot of glyceraldehyde 3-phosphate dehydrogenase (Gapdh). Expression of Gapdh did not vary much between the vehicle control group and 24 h treatment of AGN193109 with and without tRA (A), and of DEAB with and without tRA (B), evident by a tight overlapping amplification plots across all the samples. Representative amplification plots derived from technical triplicates of a single biological experiment are shown here. (TIFF) [file pone.0045725.s002.tiff]

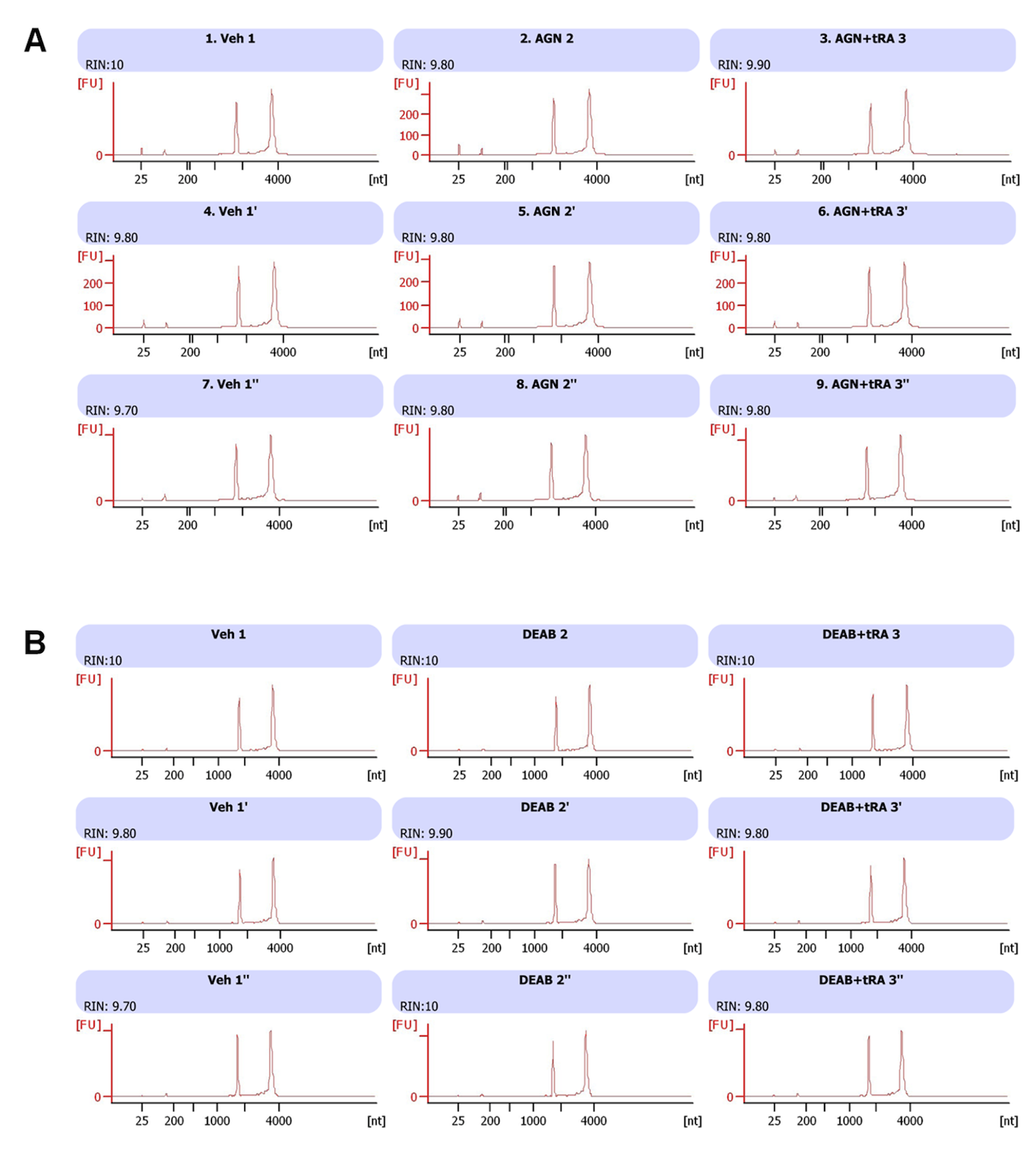

Supplement: Figure S3 — RNA integrity of samples for microarray experiments. Integrity of RNA samples from AGN193109 experiment (A) and DEAB experiment (B) was examined with bioanalyser. The two sharp major peaks correspond to 18s and 28s ribosomal RNA, respectively. The high RNA Integrity Number (RIN) values, within the range of 9.70–10.00, suggest a good quality of RNA samples with minimum degradation of RNA. There is no evidence of genomic DNA contamination in the RNA samples, given the presence of thin and sharp 18s and 28s RNA peaks, as well as absence of additional peaks other than the expected ribosomal RNA peaks. (TIFF) [file pone.0045725.s003.tiff]
